# The Metropolitan Hospital Sunday Fund

**Published:** 1905-12-23

**Authors:** 


### HOSPITAL ADMINISTRATION.

### CONSTRUCTION AND ECONOMICS.

### THE METROPOLITAN HOSPITAL SUNDAY FUND.

ANNUAL MEETING OF CONSTITUENTS.

THE VOLUNTARY HOSPITALS AND MEDICAL EDUCATION.

The annual meeting of the constituents of the Metropolitan Hospital Sunday Fund was held at the Mansion House on Monday the 19th instant, the Lord Mayor (Alderman Vaughan Morgan) presiding. There was a large attendance, the Egyptian Hall being filled and many having to stand. The proceedings at times were of an animated character. Many well-known people in the religious, medical and philanthropic world were present.

Sir Edmund Hay Currie, the Secretary, having read the minutes of the previous meeting,

The Bishop of London moved that the report of the Council for 1905 be received. That, he said, was a business meeting and not one where they had to push the cause by long speeches. But he would like to say three things. He felt that the hospitals of London were one of the grandest things they had about London at all. He had spent, as they knew, many of the best years of his life among the slums of the East End and he did not know what they would do without them. The good they did must be seen to be appreciated, and what that great city owed to the beneficence of the hospitals it was impossible to say. Then this Fund was one which drew all denominations together; they rejoiced to meet on a common platform and join in an honourable rivalry as to who could do most in the common cause of humanity. Long might it exist to draw them all in the bonds of brotherhood together. It was also a great thing that this was voluntary work, and he should be sorry if ever the hospitals came on the rates. The voluntary effort did them all good and the longer they could keep the hospitals on a voluntary basis the better he felt it would be. He must congratulate the Secretary and all concerned on the magnificent total collected that year. (Applause.)

The Rev. W. H. Harwood seconded the resolution. He said he desired, on behalf of the Free Churches, to associate himself with the gracious words of the Bishop of London as to the pleasure with which they all worked together for the common good. It was no mere platform compliment; it was a real delight to join heartily in that beneficent work. He would like, too, speaking from intimate knowledge as a member of the Council, to commend the work of the Distribution Committee and to express how much they owed to the efforts of Sir Edmund Currie. While fully appreciating the assistance given to the congregations by Mr. Herring's munificence, he hoped that its congregational character would always be the outstanding feature of the Hospital Sunday Fund.

At this point Mr. Stephen Coleridge, standing by the corner of the platform, said he wished to propose as an amendment the resolutions he had handed in.

The Lord Mayor said they could not be taken then. Mr. Coleridge pressed his point, but the Chairman adhered to his ruling, intimating, however, that the resolutions could be moved at a later stage of the proceedings. The resolution was then put and carried.

The Rev. Canon Fleming moved—

That June 17 be fixed for Hospital Sunday 1906.

For some years now, he said, there had been a happy consensus of opinion that the Sunday nearest the middle of June should be the one adopted, and that had again been chosen this year. He did not think they had yet covered the whole area available for the work. He believed that if the ministers of all denominations would, as he had made a practice of doing, begin the work four weeks ahead of Hospital Sunday by applying to members of their congregations with their own pens by means of personal letters, expressing the hope that in their happier circumstances they would send something for the help of the sick and suffering poor, they would find a response such as in his own case had had such happy results. One other point he wished to urge was that collections should also be taken in their Sunday-schools. There were at least 2,000 schools, and if each contributed but £2 10s. there would be £5,000 additional for the Fund, raised in a happy and holy way.

The Chief Rabbi said it afforded him extreme satisfaction to second the motion. He felt that it was a noble work, that brought together in brotherly love all the various denominations, pledged to do their best for the relief of pain. When King Edward's Fund and the League of Mercy were instituted there were apprehensions expressed that these new agencies might draw subscriptions away from the Metropolitan Hospital Sunday Fund. On the contrary, however, they had but served to strengthen the interest taken by the people of London in the work of the medical charities, causing them to loom more largely before their minds, and so helping further still to arouse some of the noblest and holiest emotions of which men and women could be capable.

The Dean of Carlisle, who has for many years shared with Canon Fleming the honour of making the largest collections on Hospital Sunday, moved the re-election of the Council, with the addition of the Ven. Archdeacon of Middlesex, the Rev. R. J. Campbell, the Rev. Charles Brown, the Rev. Frederick Gurdon, and Sir Frederick Treves to fill vacancies. He did so, he said, with great regret, because it signified his own severance from the Council. He had been on it many years, and had taken great interest in its work. Now by his removal to the North of England he must sever his connection, and among the many ties that had to be parted, he knew no thread it was harder to break. He could imagine no more representative names than those to be added. It was the Council's pride that it was comprehensive and undenominational. The resolution was seconded and carried.

The Lord Mayor proposed—

That the best thanks of this meeting be given to George Herring, Esq., for his generosity, which has assisted the Fund to secure the large amount of £78,379.

This was seconded by Sir William Church, who said that few knew how great an interest Mr. Herring took in the Fund. He had consented to join the Distribution Committee, and as he was a member, he could bear testimony to the value of the work he did there. The resolution was adopted with applause.

On the motion of the Rev. E. H. Pearce, seconded by Mr. F. H. Norman, it was resolved—

That the Committee of distribution be empowered to set aside a certain amount for the purpose of grants to district nursing associations, employing fully trained hospital nurses, that such amount so set aside shall not exceed 5 per cent. of the sum collected in any year, and that the laws of the constitution be altered accordingly.

The Voluntary Hospitals and Medical Education.

Mr. Coleridge then ascended the platform amidst cheers from his supporters, and moved the following resolutions :—

That the constituents of the Metropolitan Hospital Sunday Fund resolve: (1) That no hospital which makes a grant to a school from its resources for any purpose whatever shall receive any money from this fund; (2) that no hospital shall receive a contribution from this fund whose managers have invited its subscribers to transfer their subscriptions to any other object than the general funds of the hospital, until the subscriptions so transferred to any other account have been re-transferred to the general funds of the hospital.

He said he had come there not to attack the hospitals—(ironical cheers)—but to stand up for the poor, from whom in his opinion in the past, large sums of money devoted to them had been taken away and used for other purposes. Things had come to such a pass that last year King Edward's Fund found itself obliged to appoint a Commission composed of Sir Edward Fry, Lord Welby, and the Bishop of Stepney, to go into the whole question of the relations between the hospitals and their schools. They made a very careful report, with which he desired to associate himself entirely. In that report they said :

. . . The general conclusion at which we have arrived is the following: The schools confer certain considerable benefits on the hospitals, and the hospitals, by opening their doors to the students, provide a clinical laboratory and allow the students of the school to work there; this confers on them a very great benefit, because without the admission to the hospital the students could obtain little or no clinical teaching. These mutual benefits may, we think, be fairly set off the one against the other. If that be done, as we think it fairly may be, it follows that, in the case of the schools which last year received benefits in money or money's worth from the hospitals over and above the benefits last alluded to, there is no return made by the schools to the hospitals which can be treated as recouping this expenditure of the hospitals, and that (subject always to the observation made in paragraph 7) the schools still remain debtors to the hospitals in respect of these pecuniary contributions made to them.

That debt remained unpaid to that day; the benefits had been retained; and the first part of his resolution was to prevent such occurrences in the future, and to prevent any diversions whatever to medical schools. He had no doubt the meeting would pass that part of his resolution. ("No, no.") If not, then the King Edward's Fund Commission had sat in vain!

Sir Edmund Currie : It has been adopted by the Council already, and formed part of their report just moved by the Bishop of London! (Cheers and interruption.)

Mr. Coleridge said that as regarded the second part of his resolution it was addressed to certain hospitals which, seeing that the decision was against the diversion of funds, proceeded to devise means to evade the report of the Commission in a manner which they hoped would escape notice or not be understood. Two hospitals had invited their subscribers to transfer their contributions, one to a "separate" fund, the other to a "discretionary" fund, which was to be left in the hands of the managers. By this means they could still devote funds to the school which ought to go to the hospital. That he called extraordinary conduct. (Cries of "No" and "They are voluntary hospitals.") Were men, the managers of voluntary hospitals, entitled to take money away from them and give it to the medical schools? ("All of it?" "No, no." "They don't do so." "Yes they do," and much counter-cheering.)

When he could be heard above the tumult Mr. Coleridge continued : May I ask how subscribers can transfer their money to one fund without taking it away from the other? ("They don't.") I hope I speak in the presence of educated gentlemen who know their Latin and Greek. (Laughter.) If you transfer a subscription from the general fund of a hospital to a special fund you must begin by taking it away from the general fund. ("No, no.") If the gentleman says "No" he lacks logic and English. (Laughter and cheers.) Transfer means carry across.

The Rev. C. H. Grundy protested that the speaker ought to confine his remarks to the chair, and not hold conversations all over the room. This was met by further laughter and cheering, but Mr. Coleridge retorted that he was being subjected to interruptions and interrogatories from all parts of the room, and it was only human nature to reply. But he would only add that the schemes against which his resolutions were directed would, if left alone, have the effect of evading the recommendations of the Commission. ("No, no," and cheers.)

The Venerable Archdeacon of Westminster said he had great pleasure in seconding the resolutions which had been so ably, and he might say so humorously, proposed by his learned friend, and he desired to associate himself with every word he had said. The main principle that underlay the resolutions, and his object in seconding them, were not then to be alluded to—(laughter and ironical cheers)—but no subject had a tighter grip of his heart and mind, and he was prepared to fight it to the death on any other platform. He would, however, base his support of the resolutions on the commonplace ground that he was opposed to a great and honourable profession educating its alumni by means of funds contributed for the sick poor. They ought to be trained as the alumni of the other professions were. He was quite satisfied with the report of the Fry Commission, but it had been met by what looked like an attempt to override it when certain hospitals asked for a "discretionary" fund, and he personally was holding back the collection from his congregation, for he was determined that not one farthing should go for any purpose but the relief of the sick poor. If Mr. Coleridge's resolutions were not carried, Archdeacon Wilberforce said he should not hand that collection over to the Sunday Fund, but should take care that it did go to the relief of the poor.

His Grace the Rev. Archbishop Bagshawe (*in partibus*) supported the resolutions. He thought it was not honest to give money collected by congregations for the sick poor to teach young doctors the beginning of their studies. It was said that the students acted as clerks and dressers : that was true, but not till the last two years of their five or six years' course. He thought they ought to be ashamed to be taught at the cost of the sick poor. Young clerics and young lawyers were not helped in that sort of way. People would not go on subscribing to hospitals if they thought the money was going in some cases for purposes which were held in great abhorrence by very many.

The Chairman of the London Hospital—one of the hospitals referred to by Mr. Coleridge—said he doubted very much whether the resolutions proposed by Mr. Coleridge could posibly be in order. He understood that no notice had been sent to the Secretary or the Council. But he did know that a letter had gone out to the clergy all over London urging them to come there that day and so get a snap vote for Mr. Coleridge's resolutions. However, to deal with Mr. Coleridge's accusations; he declared that money contributed to the hospitals had been taken from the sick poor and given to the medical schools. But they need not think he was concerned at all for the sick poor. He was there in the interests of Anti-Vivisection. (Applause and dissent.) When he appeared to give evidence before Sir Edward Fry's Commission he admitted that he was not there in the interests of the sick poor.

Mr. Coleridge : I never said so. It is not the fact.

Mr. Holland : Well, I will read your answer to Lord Welby. You said—

I may say, personally, that I should never have raised this agitation or said a word had the schools avoided a certain practice which we object to. It is simply that that has aroused us. We feel that it is wrong that this money which is subscribed for one purpose should be diverted to this detestable practice. If only that had been omitted in the medical education given in these schools you would never have heard of Stephen Coleridge and this agitation. I do not mind telling you that frankly. If it was not for that, as far as I and those I represent are concerned, we should have left it to the hospital managers to manage as they thought best. It is that that induced me to go into this agitation.

That is not in the interests of the sick poor?

Mr. Coleridge : Read the next question.

Certainly!

938. The Chairman : Then you would rather agree that supposing this practice had been absent, you would not have seen any harm in what is being done in the administration of hospital funds?—I do not know that. I should never have found it out.

You are very easily pleased if that pleases you. But do you realise that Sir Edward Fry's Commission said in effect that money was *not* taken from the sick poor? They were asked by the King's Fund to say whether money subscribed for the relief of the sick poor to hospitals was contributed by those hospitals for the maintenance of medical education. Their answer was that so many of the schools were started before the hospitals, or both were founded together, and so much money was contributed for both that they could not answer the question how much was given for the relief of the sick poor and how much for the promotion of medical education, but begged leave to delete the words "for the relief of the sick poor" from the question, and so left in "has money given to the hospitals been used for the schools?" Turning from that subject the speaker said he did not expect to hear a bishop—or archbishop—stand up and accuse men as honourable as himself of want of honesty. Those who gave their lives to the work of the hospitals had as keen a sense of honour as those who criticised them, but they had some knowledge, and they believed it was absolutely essential that a hospital should have a good school. (Applause.) As to the recommendations of the Commission, did not everybody know that the Council of the Sunday Fund had passed the following resolutions?

I. That this Council cordially agree with paragraph 27 of the report of Sir Edward Fry's Committee appointed to inquire into the financial relations between the hospitals and medical schools, of which the following is an extract: For the future the distinction between the hospital and the school should in every case be drawn not only definitely and exactly, but with such clearness that it may be understood by the general public, and so that no question may arise as to the destination and application of moneys contributed whether by the King's Fund or from any other source.

II. That no grant shall be made from the metropolitan Hospital Sunday Fund to any hospital which fails to completely comply with the above-named recommendation.

III. That in estimating the amount to be contributed to the funds of any hospital from the Hospital Sunday Fund no moneys paid or subscribed during the preceding year towards the school attached to any hospital shall be taken into account.

(Applause.)

Those were the resolutions which had been adopted by the Council, so that those who did not want their money to go to the schools could be quite safe that not one penny would be so diverted. (Applause.)

It was not surprising that Mr. Stephen Coleridge, with his keen desire to help the hospitals—(laughter and cheers) —was annoyed at the idea of a discretionary fund. It was childish to talk about transferring subscriptions. This was for the future, and might not people so give their money if they liked? Might they not give it to the managers whom they trusted, and leave it to them to deal with as they thought best for the hospitals? How was that "not honest"? It was mere playing with words. Then as to Canon Wilberforce—he begged pardon, Archdeacon Wilberforce—(laughter)—he had Mr. Coleridge's criticism on the Archdeacon's sermon, and Mr. Coleridge wrote that what the Archdeacon said was not true. (Laughter and cheers.) The Archdeacon had told his congregation that thousands of animals had suffered prolonged torture while most of the operations had been merely inoculations, and the Blue Book stated that only a few had been seriously painful. Was that honest? ("Shame" and applause.) They knew that this agitation was not at all in the interest of the sick poor, but was concerned with the vivisection question. That might be an equally honourable motive, but it was not the one avowed now. If successful, the agitation would wreck the Sunday Fund because it would destroy confidence in it, and the subscriptions would fall off. (Applause.)

The Rev. C. H. Grundy opposed the resolutions, and warned the meeting against interfering with what, for thirty-two years, had worked so well. Their congregations were not idiots, and when they looked at the gentlemen who were in charge of the job they would not listen to Mr. Coleridge. (Laughter and cheers.)

Mr. David Christie Murray essayed to speak, but there were loud cries of "Vote," and he was not heard.

Mr. Coleridge put as a point of order that only constituents ought to vote, and that many in the hall had no right there.

Sir Edmund Currie thereupon read a letter from one of the clergy, proving that letters had been sent by the Anti-Vivisection Society asking for the tickets of admission which had been issued to the clergy to be sent to the Secretary to admit the Society's nominees to that meeting.

The Lord Mayor then put the resolutions separately, and declared them lost. The majority against the resolutions was overwhelming.

Mr. Coleridge claimed a scrutiny, but this was not acceded to. In fact, every ticket was scrutinised on presentation, so that the meeting was confined to those who were entitled to be present.

The Venerable Archdeacon of London then rose, amid loud applause, to propose a vote of thanks to the Lord Mayor, who had, he said, earned their gratitude by his able, kind, and conciliatory conduct in the chair.

Sir Savile Crossley seconded, and it was carried with cheers.

